# Circumferential Crack Detection in Ultra-High-Pressure Tubular Reactors with Pulsed Eddy Current Testing

**DOI:** 10.3390/s24206599

**Published:** 2024-10-13

**Authors:** Yaxing Wang, Jie Sun, Huasheng Hu, Bo Hu, Weiqi Bin, Wen Shi, Yuewen Fu

**Affiliations:** 1 Key Laboratory of Nondestructive Testing, Ministry of Education, Nanchang Hangkong University, Nanchang 330063, China; wangyaxing203@163.com (Y.W.); cumthubo@163.com (B.H.); bwq202202@163.com (W.B.); shiwen2727@163.com (W.S.); 2 Guangdong Institute of Special Equipment Inspection and Research, Foshan 528251, China; sunjie@gdsei.org.cn (J.S.); huashenghu@126.com (H.H.)

**Keywords:** pulsed eddy current, ultra-high-pressure tubular reactor, cracks, lift-off, eddy current distribution

## Abstract

Ultra-high-pressure tubular reactors are crucial pieces of equipment for polyethylene production. Long-term operation under high temperature, high pressure, and other extremely harsh conditions can lead to various defects, with circumferential cracks posing a major safety risk. Detecting cracks is challenging, particularly when they are under a protective layer of a certain thickness. This study designed a pulsed eddy current differential probe to detect circumferential cracks in ultra-high-pressure tubular reactors, with the lift-off distance acting as a protective layer. Detection models for traditional cylindrical and semi-circular excitation differential probes were established using finite element simulations. Corresponding experiments under different lift-off conditions were carried out, and the model’s accuracy was verified by the consistency between the simulation results and experimental data. The distribution of the eddy current field under different conditions and the disturbances caused by cracks at various positions to the detection signal were then calculated in the simulations. The simulation results showed that the cracks significantly disturbed the eddy current field of the semi-circular excitation differential probe compared with that of the traditional cylindrical probe. The designed differential probe effectively detected circumferential cracks of specific lengths and depths using the difference in the voltage signals. The experimental results were in agreement with the simulation results, showing that the designed probe could effectively detect 20 mm-long circumferential cracks at a lift-off of 60 mm. The experimental results also show that the probe’s detection coverage area in the axial direction varied with the lift-off height. The probe design and findings are valuable for detecting cracks in ultra-high-pressure tubular reactors with protective layers.

## 1. Introduction

Polyethylene is extensively used in industries such as textiles, petrochemicals, plastics, and automobiles due to its low cost and excellent chemical properties, leading to a steadily increasing market demand [1]. Ultra-high-pressure tubular reactors are essential in polyethylene production, typically operating at internal pressures of 100–300 MPa and temperatures around 300 °C. The medium typically consists of ethylene and high-pressure polyethylene. Due to severe operating conditions such as high temperatures and pressures, the pipelines inevitably suffer various types of damage. The explosive nature of the medium means that any rupture could result in catastrophic accidents and heavy losses [2]. Currently, there are no explicit requirements for the periodic inspection of ultra-high-pressure tubular reactors in China. Most studies on ultra-high-pressure reactors have focused on self-enhancing residual stress and the fatigue characteristics of tubular reactors [3,4], and there is a lack of convenient and reliable methods for detecting the most dangerous defects, such as cracks.

Ultra-high-pressure tubular reactors used in polyethylene production typically have a casing tube structure, where the inner tube bears the ultra-high-pressure load. The most severe defects, such as cracks, often occur on the outer wall of the inner tube. However, older ultra-high-pressure tubular reactors have different structures, usually a single-layered tube under an insulation layer. Structural damage is inevitable during operation despite the protection. Traditional methods such as eddy current, ultrasound, and electromagnetic ultrasound require removing the reactor’s insulation layer or even grinding the pipe surface, resulting in high detection costs and low efficiency [5,6]. Pulsed eddy current detection, due to its non-contact and wide-frequency characteristics, has been widely applied in inspecting special equipment and aerospace devices. It can be used to inspect pipelines without removing insulation and has become an advanced non-destructive testing technology in the pipeline industry [7,8,9]. A pulsed eddy current is highly sensitive to surface and deep defects and can be used to inspect ferromagnetic pipelines [6,10,11]. The excitation source of a pulsed eddy current is a square wave, decomposed into various frequencies via Fourier transformation, allowing for detection of defects at different depths [12,13]. The high-pressure conditions of ultra-high-pressure reactors result in much thicker pipeline walls than regular pipelines, making crack detection particularly challenging, especially when a protective layer is present.

Most studies on pulsed eddy current detection have focused on wall thickness measurements, while crack detection has primarily been studied under conditions with little or no lift-off [14,15,16,17,18]. Crack detection in ferromagnetic materials with some lift-off has not been thoroughly studied. Therefore, this study focuses on detecting circumferential cracks in ultra-high-pressure tubular reactors with a protective layer under specific lift-off conditions. Numerical simulation models for both differential and absolute probes were established, and their eddy current fields in detecting circumferential cracks were compared. For the differential probe, disturbances caused by cracks at different positions to the detection signal were analyzed through simulations, and the probe’s effective axial coverage area during detection was studied. Finally, the designed probe was experimentally tested, and the results were compared with those of the simulations. In practical experiments, the differential probe effectively detected circumferential cracks in ultra-high-pressure tubular reactors under specific lift-off conditions.

## 2. PECT Method and Experimental Platform

### 2.1. PECT Method

**Figure 1:**
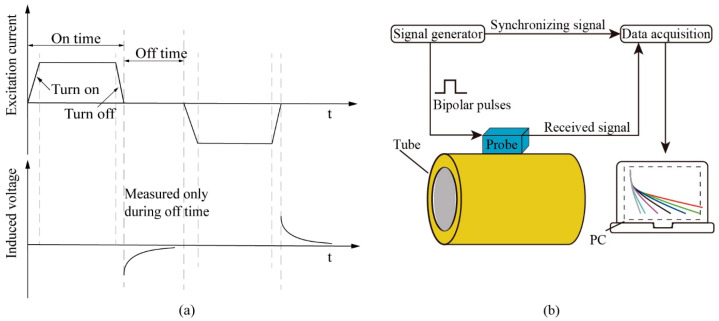
Pulsed eddy current detection principles and system schematic: (**a**) principle diagram; (**b**) system diagram.

Pulsed eddy currents typically use low-frequency square-wave excitation. When the excitation current is turned off, the primary magnetic field induced by the excitation coil rapidly decays, generating instantaneous eddy currents in the test object. As the eddy currents decay, they generate a magnetic field that opposes the primary magnetic field. This secondary magnetic field carries information about the test object’s wall thickness and defects. Processing the induced voltage signals of the eddy current magnetic field reveals various defect details in the specimen. The principles and a system schematic of pulsed eddy current detection are shown in Figure 1.

### 2.2. Experimental Platform and Specimens

**Figure 2:**
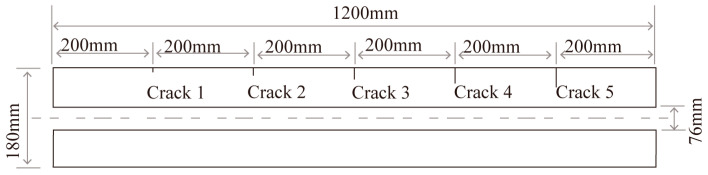
Schematic of pipe dimensions.

**Figure 3:**
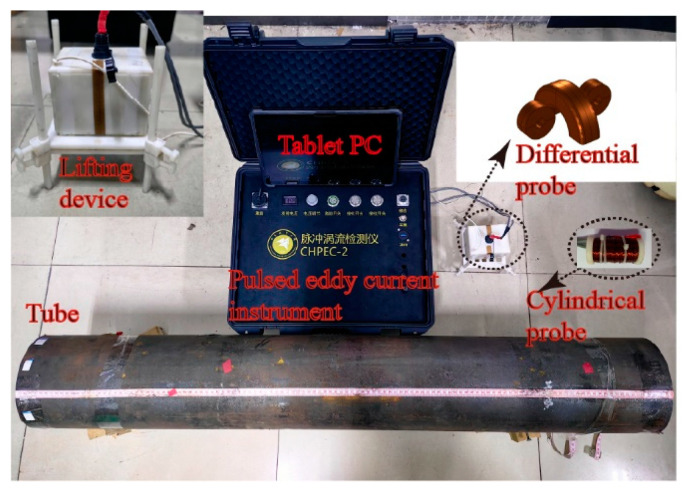
Experimental platform for PECT.

The experimental platform comprised a lab-developed pulsed eddy current instrument, a simulated sample tube, probes, and auxiliary devices. In the experiment, the pulsed eddy current excitation was set to 3 A, with a 50% square-wave duty cycle and a frequency of 4 Hz. The excitation currents were selected to obtain enough signal intensity to ensure detection sensitivity while avoiding higher temperatures of the coils. The excitation duty cycle is chosen to increase the strength of the received signal [19]. A low excitation frequency is used to improve the penetration depth of PEC to detect and distinguish the deep defects. After several experiments, a square wave excitation frequency of 4 Hz was verified as the appropriate frequency. The experimental pipe was made of 42CrMo steel, with a wall thickness of 52 mm. Five circumferential cracks, each 20 mm long with various depths of 5 mm, 10 mm, 15 mm, 20 mm, and 25 mm, were machined onto the pipe’s outer wall. The pipe’s dimensions are shown in Figure 2, and the probe parameters in the experiment match those used in the simulation. The experimental platform setup is shown in Figure 3.

## 3. Simulation Modeling and Validation

### 3.1. Setup of Simulation Models

**Figure 4:**
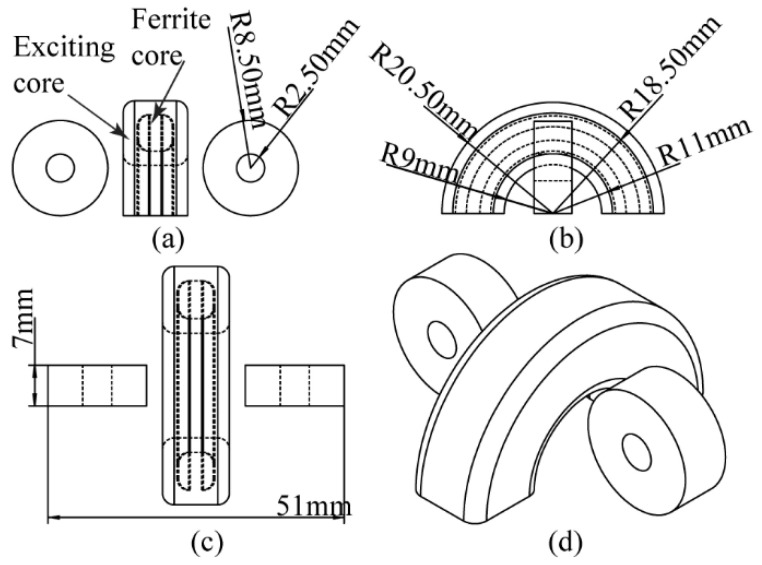
Differential probe structure: (**a**) front view; (**b**) side view; (**c**) top view; (**d**) overall view.

**Figure 5:**
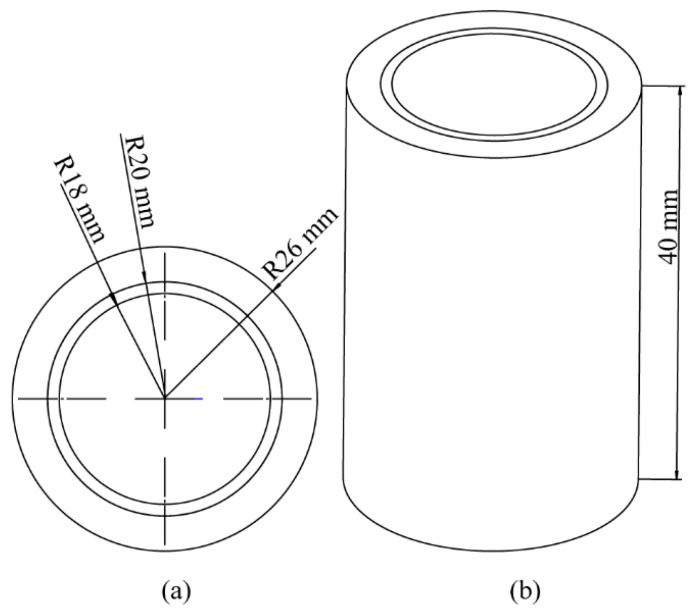
Coaxial cylindrical probe structure: (**a**) top view of probe; (**b**) overall view of probe.

**Figure 6:**
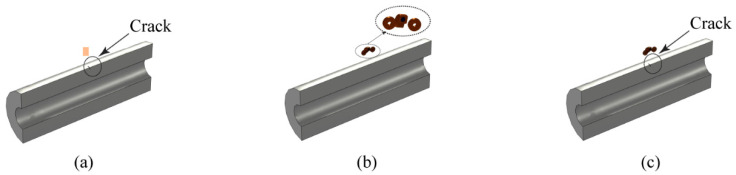
Simulation models: (**a**) traditional probe detection model; (**b**) differential probe detection model without defects; (**c**) differential probe detection model with crack defects.

A half-model with a refined grid was used in this study to improve calculation accuracy while maintaining computational efficiency. First, a pipe with cracks was constructed in the finite element simulation model. The pipe measured 520 mm in length, 76 mm in inner diameter, and 52 mm in wall thickness, with a crack located on its outer wall at the center. The crack measured 20 mm in length and 5 mm in depth. Next, traditional coaxial cylindrical probe and semi-circular differential probe models were established in the simulation. The semi-circular differential probe consisted of a semi-circular excitation coil with a magnetic core and two non-core receiving coils, as shown in Figure 4. The traditional coaxial cylindrical probe used for comparison is shown in Figure 5, with the excitation coil wound around the receiving coil’s exterior. The probe parameters in the model match those from the actual experiment. Figure 6 presents the half-models of the pipes and probes with and without defects.

Taking the eddy current’s skin effect into account, five boundary layers were added to the pipe’s outer surface during mesh subdivision to enhance the eddy current’s resolution and improve calculation accuracy. The simulation model used a 3 A excitation current and a 4 Hz pulsed excitation frequency, consistent with the experimental parameters. The simulated sample pipe was made of 42CrMo steel, with a relative magnetic permeability of 180 and an electrical conductivity of 4.676 × 10⁶ S/m.

### 3.2. Modeling Validation

**Figure 7:**
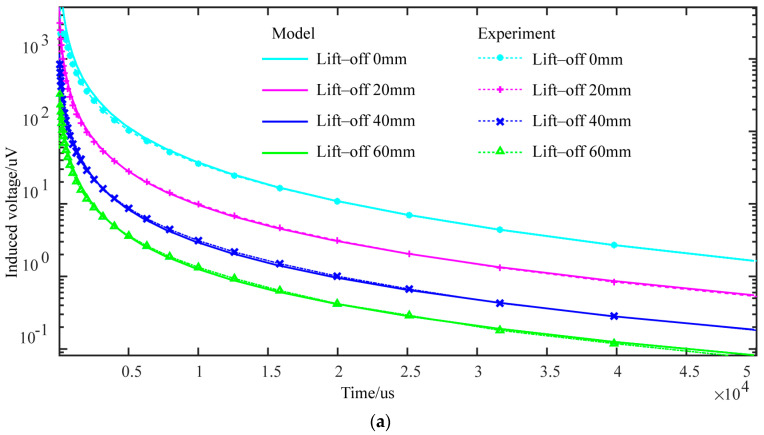

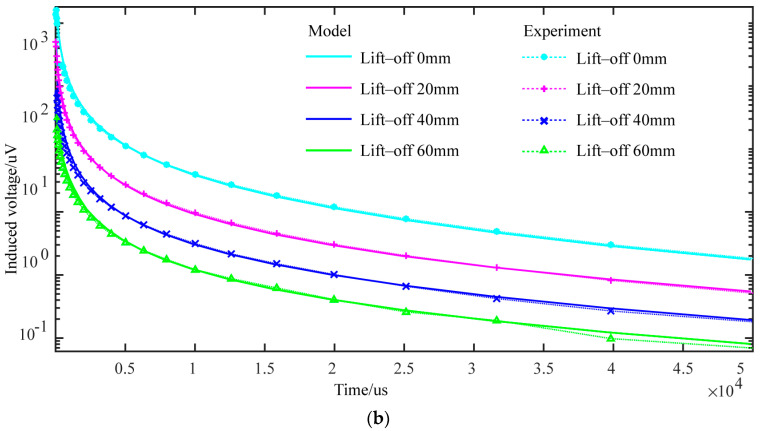
Comparison of experimental and simulated voltage decay curves for a single receiving coil at different lift-offs: (**a**) defect-free pipe; (**b**) pipe with 5 mm-deep crack.

To verify the model’s accuracy, the numerical simulation results of the semi-circular differential probe at different lift-offs were compared with the actual experimental results of a defect-free pipe. Figure 7 compares the experimental voltage decay curves with the numerical simulation results at lift-offs of 0 mm, 20 mm, 40 mm, and 60 mm. The significant overlap between the simulation and experimental results confirms the accuracy of the numerical simulation model used in this study.

## 4. Simulation Results

### 4.1. Perturbation Analysis of Eddy Currents by Cracks under Conventional Cylindrical Probe Excitation

**Figure 8:**
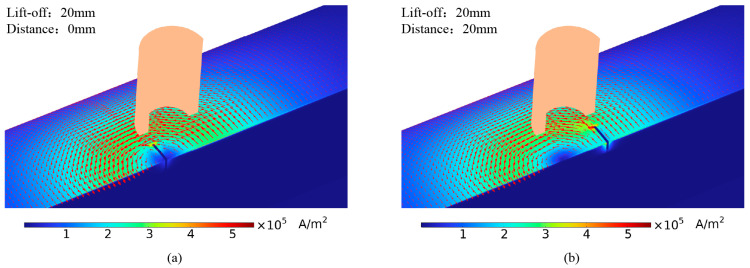

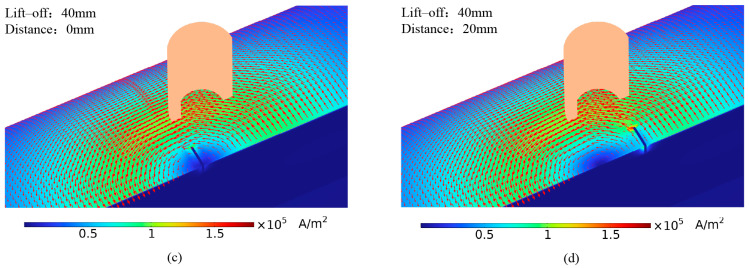
Eddy current distribution with cylindrical probe at different distances from crack: (**a**) lift-off 20 mm, probe and crack distance 0 mm; (**b**) lift-off 20 mm, probe and crack distance 20 mm; (**c**) lift-off 40 mm, probe and crack distance 0 mm; (**d**) lift-off 40 mm, probe and crack distance 20 mm.

Figure 8 illustrates the eddy current distribution on the pipe surface when the crack is at different distances from the probe center, with lift-offs of 20 mm and 40 mm. As shown in Figure 8, the eddy current density directly beneath the probe is almost zero, indicating a detection blind spot in the traditional cylindrical probe. When the crack is directly under the probe, the eddy current distribution on the pipe remains largely unaffected due to the blind spot. When the crack is not directly beneath the probe, the induced eddy currents on the pipe flow tangentially to the crack, causing less significant disturbances to the eddy current flow. As lift-off increases, the eddy current’s divergence on the pipe surface also increases, lowering the crack detection sensitivity.

Due to the blind spots and other limitations of traditional cylindrical probes, this study developed a semi-circular excitation probe. The semi-circular probe induces more concentrated eddy currents in the pipe, eliminating the detection blind spot and partially mitigating the eddy current divergence caused by lift-off. Adjusting the semi-circular probe placement aligns the eddy current flow in the pipe perpendicular to the crack, enhancing its disturbance of the eddy current flow.

### 4.2. Perturbation of Eddy Current Field and Received Signal by Cracks under Semi-Circular Excitation Probe

**Figure 9:**
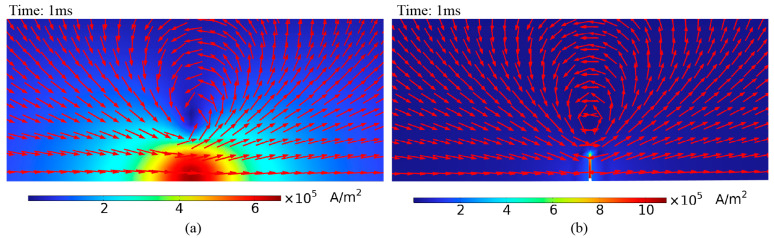
Distribution of eddy currents on the top surface of the pipe: (**a**) defect-free pipe; (**b**) pipe with crack.

**Figure 10:**
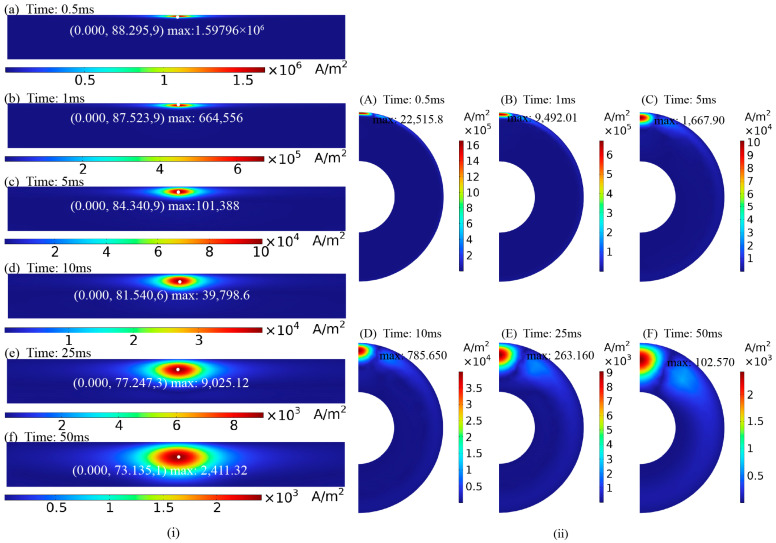
Eddy current distribution in a defect-free pipe cross-section at different moments: (**i**) axial cross-section; (**ii**) cross-section of the pipe wall.

Figure 9 shows the eddy current distribution on the pipe surface when detected using the semi-circular probe. Without a crack, the eddy currents on the pipe surface circulate around the top and converge directly beneath the probe, where the eddy current density is the highest, eliminating the detection blind spot. Figure 10 shows the eddy current distribution over time as it penetrates the middle region of the pipe wall. The eddy currents on the surface of the pipe flow parallel to the axial direction under the semi-circular probe, allowing the receiving coil to be placed parallel to the pipe axis (as shown in Figure 4 and Figure 6b) to maximize the received eddy current magnetic field signal.

**Figure 11:**
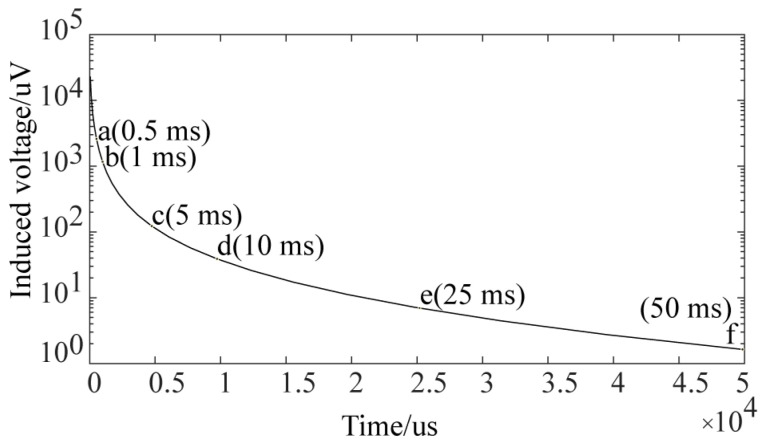
Simulated voltage decay curve of receiving coil without defects (corresponding to eddy current distribution in Figure 10).

Figure 11 shows the voltage signal of a single receiving coil in a defect-free pipe under semi-circular probe excitation. The voltage signal can be broken down into three stages. The first stage reflects the voltage induced by the combined effects of the excitation magnetic field and eddy current magnetic field after the excitation current is turned off (before point b). The second stage corresponds to the period when the eddy currents penetrate the middle of the pipe wall (from points b to f). The third stage shows the period when the eddy currents diffuse toward the ends of the pipe (after point f). The eddy current distribution corresponding to the voltage decay curve at different times is shown in Figure 10.

**Figure 12:**
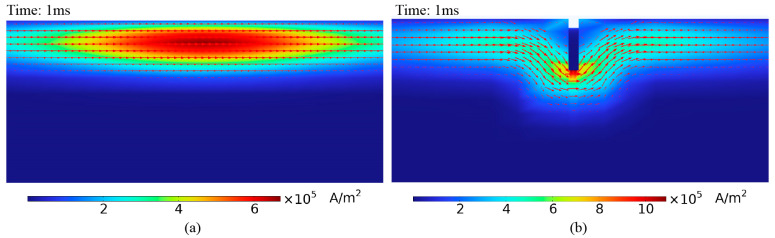
Distribution of eddy currents in the axial cross-section of the pipe: (**a**) defect-free pipe, (**b**) pipe with crack.

**Figure 13:**
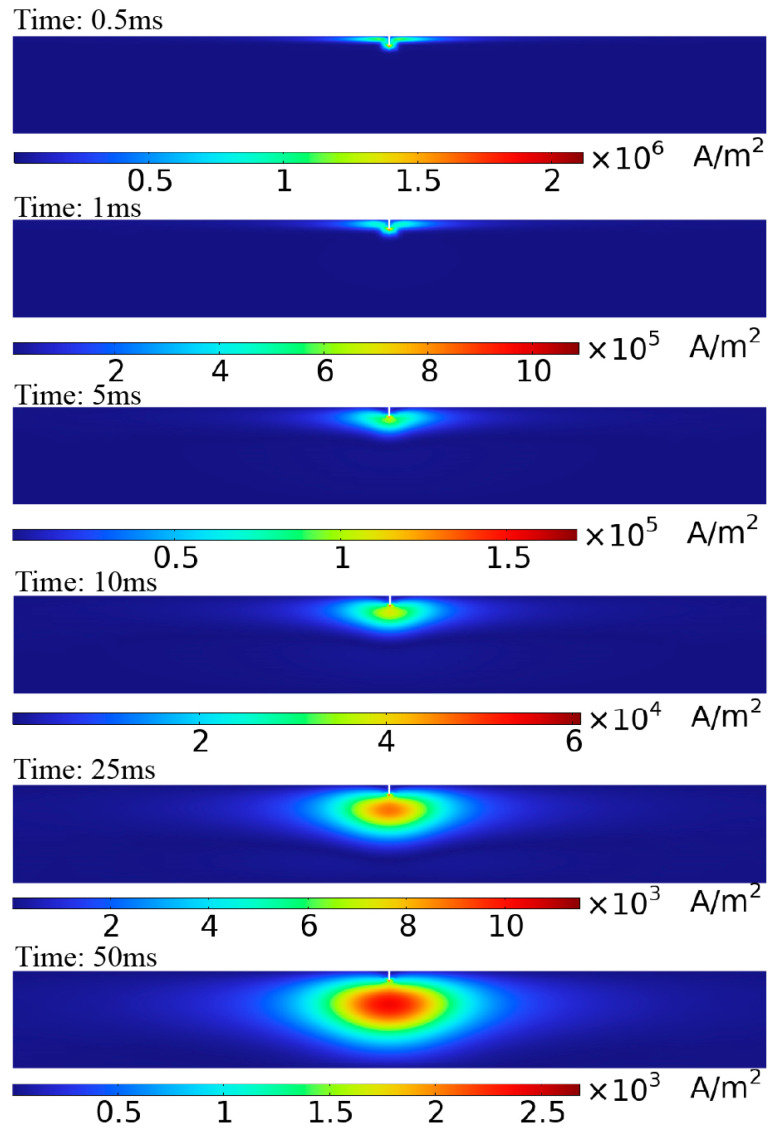
Distribution of eddy currents at different moments in the axial section of defective pipe.

**Figure 14:**
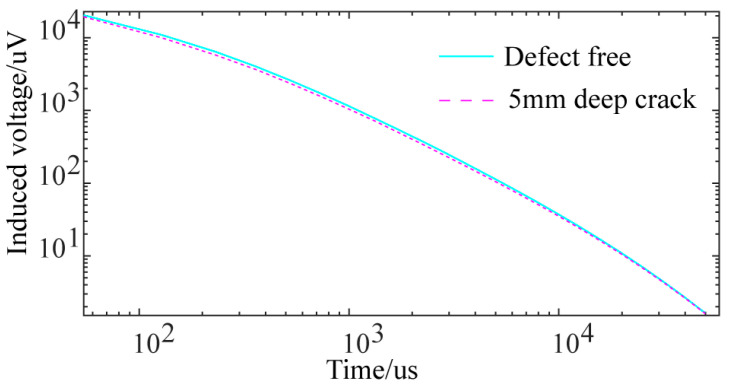
Simulation of voltage decay curves of a single receiving coil for pipes with and without defects.

When a defect is present under the semi-circular excitation probe, the eddy current distribution is disturbed, as shown in Figure 9b, Figure 12b and Figure 13. The eddy currents flow around the defect, altering the magnetic field that they induce, which disturbs the voltage signal in the receiving coil. Figure 14 presents the voltage signals from a single receiver coil located close to the crack. The voltage signal decays more rapidly in the early stages when a defect is present than when there is no defect. As shown in Figure 13, surface crack defects have a more significant impact on the early eddy current distribution, resulting in a greater influence on the early part of the voltage decay curve.

### 4.3. Simulation Analysis of Crack Detection Signals at Different Positions in the Detection Area of the Semi-Circular Probes

**Figure 15:**
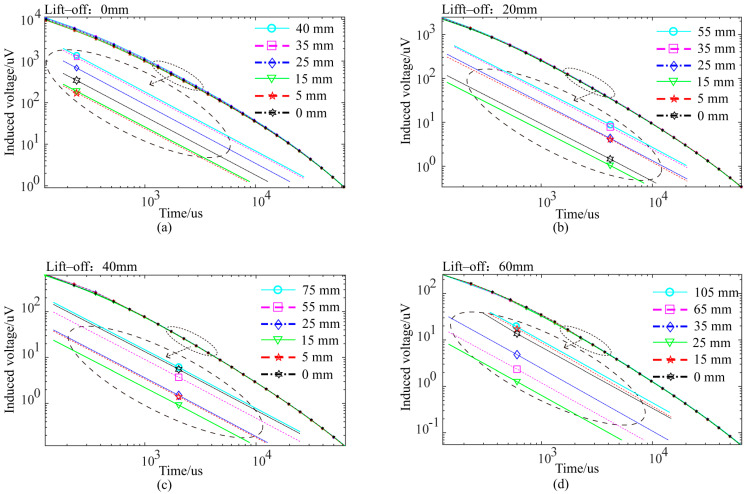
Voltage decay curves at different distances between crack and probe center: (**a**) lift-off 0 mm; (**b**) lift-off 20 mm; (**c**) lift-off 40 mm; (**d**) lift-off 60 mm.

Figure 15 shows the voltage decay curves of a single receiving coil at different horizontal distances from the center of the semi-circular excitation coil. The voltage decay curves show that the disturbance caused by the crack to the received signal varies with the crack’s horizontal position relative to the probe. The disturbance is the greatest when the crack is directly beneath the receiving coil.

Without lift-off, when the distance between the probe’s excitation coil center and the crack exceeds 40 mm, the receiving coil’s voltage signal curves are coincident (a difference smaller than 1 microvoltage is considered unrecognizable). Cracks within 40 mm of the probe’s excitation coil center are detected by the receiving coil. In the simulations, a voltage signal difference greater than 1 microvoltage caused by a defect is considered distinguishable, and the defect is detected, as a microvolt-level signal difference can be recognized by the actual PEC equipment used in this study.

**Figure 16:**
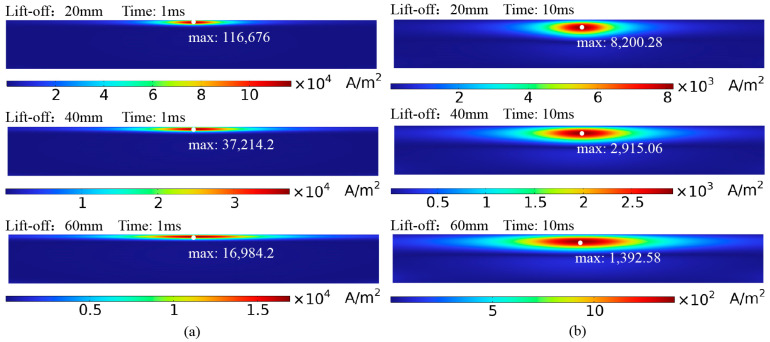
Eddy current distribution in the axial cross-section of the pipe at different lift-off heights: (**a**) 1 ms; (**b**) 10 ms.

Figure 16 shows the eddy current distribution in the axial cross-section of the pipe at lift-offs of 20 mm, 40 mm, and 60 mm at various time points. As the lift-off increases, the concentrated eddy current area within the pipe expands, the eddy current density decreases, and the amplitude of the voltage decay curve decreases (as shown in Figure 7). As the lift-off height increases, the probe’s detection coverage area also expands due to the divergence of the magnetic field induced by the excitation.

Therefore, the probe’s effective detection area changes with the lift-off. Figure 15 shows that the perturbation of eddy currents caused by cracks can be recognized in the voltage signals when the probe lift-off is set to 20 mm, 40 mm, or 60 mm and the transverse distance between the crack center and the probe center is smaller than 55 mm, 75 mm, or 105 mm, respectively.

## 5. Experimental Results

### 5.1. Signal Processing Method

The decay rates of voltage decay curves for positions with defects differ from those of the voltage decay curves in defect-free area, owing to the disturbances to the eddy current and magnetic field caused by the defects [14]. In this study, the time slice method was used to show the difference among the decay rates of the voltage in the receiving unit for different measured points. The following is the detailed description of data processing performed using the time slice method.

During detection, the probe was moved along the pipe’s axial direction, and detection points were selected every few millimeters. For the voltage values of the receiving coil, let M represent the number of detection points, and let N represent the number of sampling time points for each detection point. The data for the m-th detection point can be represented by a vector:

(1) $$\underset{X_{m}}{\to }=\left[x_{m1},\;x_{m2},\;\cdots ,\;x_{mN}\right]$$

The data for all M detection points can be expressed in matrix form:

(2) $$X=\left[\begin{array}{c} \begin{array}{c} \vec{X_{1}}\\ \vec{X_{2}} \end{array}\\ \vdots \\ \vec{X_{M}} \end{array}\right]=\left[\begin{array}{ccc} \begin{array}{cc} x_{11} & x_{12}\\ x_{21} & x_{22} \end{array} & \cdots & \begin{array}{c} x_{1N}\\ x_{2N} \end{array}\\ \vdots & \ddots & \vdots \\ \begin{array}{cc} x_{M1} & x_{M2} \end{array} & \cdots & x_{MN} \end{array}\right]$$

**Figure 17:**
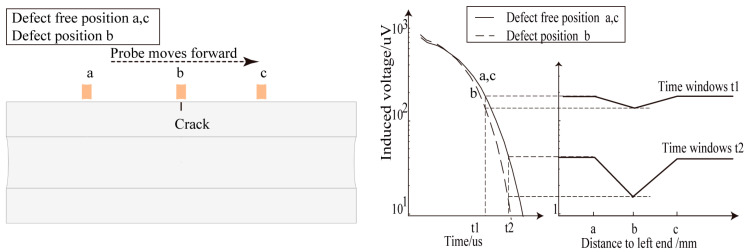
Characteristic signal of the defect in time slice curves of traditional cylindrical probe.

**Figure 18:**
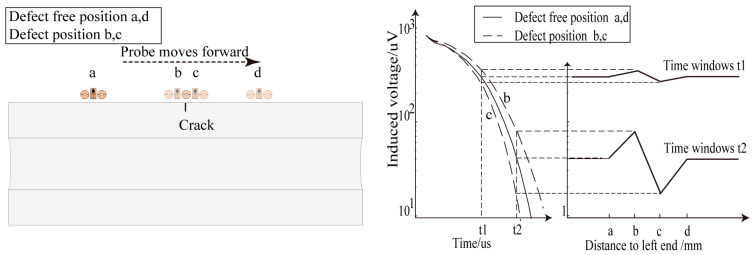
Characteristic signal of the defect in time slice curves of differential probe.

Here, the row vectors of matrix *X* represent the induced voltage values of each detection point at different sampling times, and the column vectors represent the induced voltage values of each time point across all detection points. A curve is plotted with the detection point coordinates on the horizontal axis and the column vector of the same time point on the vertical axis. This curve is referred to as the detection voltage decay in time slice curve at a specific time point. Figure 17 and Figure 18 show examples of the voltage decay in time slice curves using a traditional cylindrical absolute probe and the designed pulsed eddy current differential probe, respectively. The horizontal axis represents measured points, and the vertical axis represents voltages in the receiving unit at different time intervals: t1, t2. In the voltage decay in time slice curves, if there is a defect at a detection point, the induced voltage at that point will show an anomaly. In Figure 17, using the traditional cylindrical absolute probe, a “V”-shaped waveform appears at the defect location. In Figure 18, using the designed pulsed eddy current differential probe, a sine-wave pattern appears at the defect location. These distinct patterns allow for defect identification. Compared with absolute signals, differential signals can reduce the effects of the excitation magnetic field and noise and provide higher detection sensitivity for small defects.

In this study, the entire detection signal sampling window is divided into 31 time windows with logarithmically spaced intervals. As the voltage signal from the receiving coil decayed approximately at logarithm law, the length of the windows increased logarithmically. The information in the signal could be better retained in this dividing way in the subsequent signal processing method [20]. The center times of each window are listed in Table 1.

### 5.2. Experimental Design

In this experiment, the semi-circular excitation probe was used, with the two symmetrically placed receiving coils connected in differential mode to minimize the impact of the excitation magnetic field on the received signal. This configuration also reduces the influence of noise during detection. The structure and placement method of the semi-circular excitation probe are shown in Figure 4.

**Figure 19:**
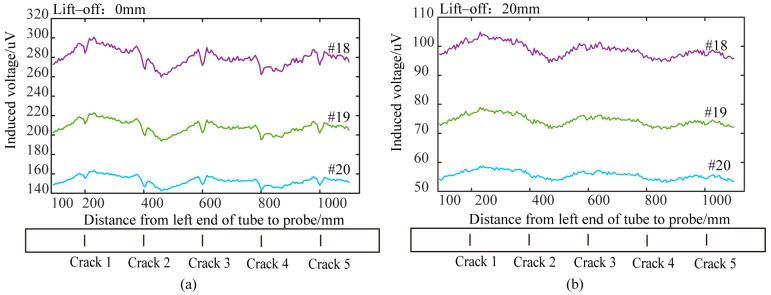
Detection results of traditional cylindrical probe at different lift-off heights: (**a**) lift-off 0 mm; (**b**) lift-off 20 mm.

**Figure 20:**
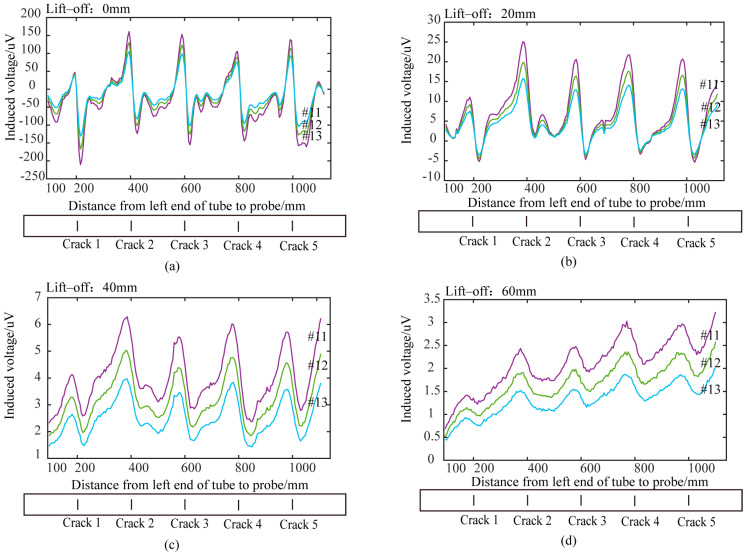
Detection results of differential probe at different lift-off heights: (**a**) lift-off 0 mm; (**b**) lift-off 20 mm; (**c**) lift-off 40 mm; (**d**) lift-off 60 mm.

Traditional cylindrical absolute probes and semi-circular differential probes (with two differentially connected receiving coils) were tested on the pulsed eddy current platform described in Section 2. Circumferential cracks of various depths on the 42CrMo pipe were scanned at equal intervals (5 mm) under different lift-off conditions. Figure 19 and Figure 20 show the detection results of circumferential cracks with depths of 5 mm (Crack 1), 10 mm (Crack 2), 15 mm (Crack 3), 20 mm (Crack 4), and 25 mm (Crack 5) at lift-offs of 0 mm, 20 mm, 40 mm, and 60 mm.

### 5.3. Analysis of Experimental Results of Conventional Cylindrical Absolute Probe

Using the method described in Section 5.1 to process the signals from the absolute probe, time slice curves corresponding to the time windows 18, 19, and 20 were extracted, and the “V”-shaped waveforms were used to identify the circumferential cracks. Figure 19 shows the detection results of the different crack depths at lift-offs of 0 mm and 20 mm. The results show that, at zero lift-off, the traditional absolute probe could detect the circumferential cracks on the ultra-high-pressure tubular reactor by recognizing the “V”-shaped waveform in the time slice curves. However, at a lift-off height of 20 mm, the traditional cylindrical probe could no longer effectively detect the cracks. Since the eddy current induced by the conventional probe is not perpendicular to the crack, it is not significantly disturbed. Additionally, the eddy current becomes more diffuse and weaker as the lift-off increases. Therefore, the traditional probe cannot effectively detect circumferential cracks on the outer wall of ultra-high-pressure tubular reactors with protective layers.

### 5.4. Analysis of Experimental Results of Differential Probes with Semi-Circular Excitation

Using the signal processing method for differential probes described in Section 5.1, the differential voltage signals from the semi-circular excitation differential probe were processed. Figure 20 shows time slice curves for circumferential cracks at different lift-off heights. Time slice curves for the time windows 11, 12, and 13 were extracted, and the sine-wave patterns were used to identify the cracks. Cracks with depths of 5 mm or more could be accurately detected based on the sine-wave pattern within a lift-off range of 60 mm. Compared with traditional cylindrical probes, the semi-circular differential probe could overcome lift-off effects and detect cracks of various depths more effectively. Therefore, the designed semi-circular pulsed eddy current differential probe showed superior performance in detecting cracks to traditional cylindrical probes.

Figure 20 shows that, under the same detection conditions, the probe detects a 5 mm crack less effectively than a deeper crack. Figure 12b indicates that when a crack appears, the eddy current bypasses it. The deeper the crack, the further the eddy current is diverted, leading to greater disturbance and a more obvious detection effect.

**Figure 21:**
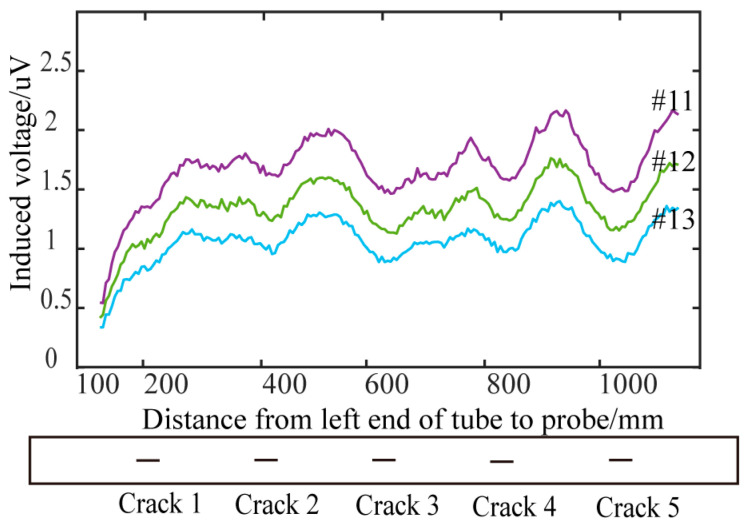
Detection results of the differential probe for axial crack at lift-off 60 mm.

To assess the applicability of the semicircular excitation differential probe for detecting axial cracks, the probe was used to detect outer wall axial cracks with a length of 20 mm and depths of 5 mm, 10 mm, 15 mm, 20 mm, and 25 mm. The results of axial crack detection at different depths with a 60 mm lift-off height are shown in Figure 21. Figure 21 shows that axial cracks with depths of 5 mm and 10 mm were not detected at 60 mm lift-off height. Lower voltage amplitude in time slices of axial crack detection results compared to circumferential crack detection results. This occurs because the axial crack causes less eddy current disturbance than the circumferential crack under the same detection conditions. Therefore, the semicircular differential probe has lower detection capability for axial cracks compared to circumferential cracks. The semicircular differential probe can be rotated 90° horizontally for effective detection of shallow circumferential cracks. Thus, two perpendicular semicircular differential probes can simultaneously achieve effective detection of both circumferential and axial cracks.

Finally, the detection coverage area of the semi-circular differential probe along the pipe’s axial direction was analyzed. The experimental results in Figure 20 show that, when scanning a pipe with five circumferential cracks of various depths spaced 200 mm apart, the pipe edges interfered with the detection signals when the distance between the probe center and the pipe edge was less than 150 mm. For lift-offs of 0 mm and 20 mm, cracks within 45 mm and 60 mm of the excitation center were detected. However, for lift-offs of 40 mm or 60 mm, the horizontal sections between adjacent cracks disappeared from the voltage time slice curves, indicating that the detection of non-defective areas is affected by interference from nearby cracks. Additionally, at a lift-off of 60 mm, the amplitude of the voltage signal in the no-defect regions gradually increased. This increase was attributed to the expanded detection area caused by the increased lift-off, resulting in signal interference from adjacent cracks. The calculated detection area from the simulation was consistent with the experimental results. Table 2 presents the axial detection coverage areas for the designed differential probe at various lift-off heights.

**Figure 22:**
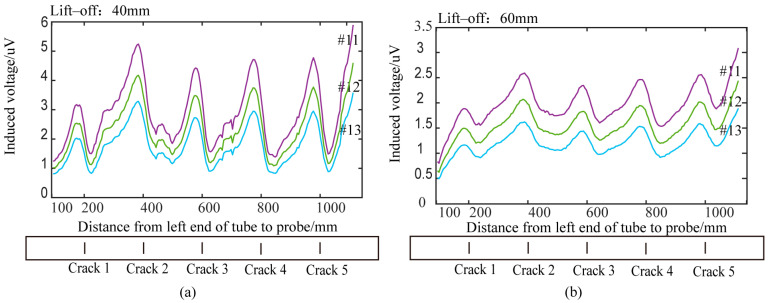
Detection results of differential probe at excitation frequency of 32 Hz: (**a**) lift-off 40 mm; (**b**) lift-off 60 mm.

To analyze the effect of excitation frequency on the coverage of detection along the pipe’s axial direction, the semicircular differential probe was used with an excitation frequency of 32 Hz to detect circumferential cracks of varying depths at different lift-offs. The results are shown in Figure 22. For lift-offs of 40 mm and 60 mm, the crack of 5 mm depth within 45 mm and 60 mm of the excitation center were detected. A reduced axial detection area was found compared to the 4 Hz excitation frequency. Different excitation frequencies result in varying eddy current diffusion time and distributions, causing changes in the detection coverage area.

## 6. Conclusions and Future Work

This study investigated the pulsed eddy current detection of circumferential cracks in ultra-high-pressure tubular reactors under specific lift-off conditions using numerical simulations and experiments. A finite element numerical simulation model was established, and its accuracy was verified by comparing the simulation results with experimental data using a semi-circular excitation probe. Simulation models for the traditional cylindrical probe and the designed semi-circular probe were constructed, and the disturbances caused by cracks to the eddy current flow were analyzed. The results show that the semi-circular probe overcame the cylindrical probe’s detection blind spots, and the disturbance to the eddy current was the greatest when the eddy current flow was perpendicular to the crack. Through a numerical analysis, reception signals at different crack positions relative to the probe were calculated, and the axial detection coverage area of the designed semi-circular probe was analyzed. The designed probe was subsequently tested in actual experiments. The experimental results matched the simulation results, showing that circumferential cracks of 5 mm or deeper in 52 mm thick 42CrMo ultra-high-pressure tubular reactors could be detected accurately with the designed pulsed eddy current differential probe at lift-offs of 60 mm or less. Additionally, the detection performance of the differential probe surpassed that of the traditional cylindrical probe under lift-off conditions. This study offers a valuable reference for the pulsed eddy current detection of circumferential cracks in ultra-high-pressure tubular reactors with cladding, and it holds significant engineering application value.

Additionally, the simulation results suggest that the duration of the eddy current disturbance caused by cracks varies with crack depth. The deeper the crack, the longer the eddy current disturbance in the pipe, resulting in a longer disturbance to the voltage signal in the receiving coil. Therefore, the crack depth can be quantified by analyzing the duration of voltage signal disturbance.

Future work will involve quantifying crack depth and further improving probe sensitivity by optimizing the probe structure. The applicability of the probe in detecting other types of defects will also be investigated.

## Figures and Tables

**Table 1:** Signal window division.

Window Number	Center Time (μs)	Window Number	Center Time (μs)	Window Number	Center Time (μs)	Window Number	Center Time (μs)
1	6	9	335	17	2011	25	12,598
2	46	10	414	18	2528	26	15,856
3	79	11	518	19	3172	27	19,965
4	104	12	645	20	3990	28	25,131
5	135	13	804	21	5024	29	31,632
6	176	14	1010	22	6319	30	39,817
7	215	15	1273	23	7956	31	50,117
8	271	16	1598	24	10,015		

**Table 2:** Axial detection coverage area of semi-circular differential probe.

**Lift-Off Height**	0 mm	20 mm	40 mm	60 mm
**Detection Area**	45 mm	60 mm	75 mm	>100 mm

## Data Availability

Data are contained within the article.
